# The Implementation of a Clinical Ladder in Rural Japanese Nursing Education: Effectiveness and Challenges

**DOI:** 10.3390/healthcare9040469

**Published:** 2021-04-15

**Authors:** Satoko Maejima, Ryuichi Ohta, Chiaki Sano

**Affiliations:** 1 Department of Nursing, Unnan City Hospital, 96-1 Iida, Daito-cho, Unnan City 699-1221, Japan; satoko.m.lynette@gmail.com; 2 Community Care, Unnan City Hospital, 96-1 Iida, Daito-cho, Unnan City 699-1221, Japan; 3 Department of Community Medicine Management, Faculty of Medicine, Shimane University, 89-1 Enya cho, Izumo 693-8501, Japan; sanochi@med.shimane-u.ac.jp

**Keywords:** clinical ladder, nursing education, rural hospitals, self-assessments, Japanese culture

## Abstract

The clinical ladder is an essential tool for nursing education, enabling nurses to ascend from novice to expert. The learning content for nurses can depend on their clinical situations. The aging of societies has changed the demand for nurses at community hospitals because of the multimorbidity of older patients. At the same time, the gap in nursing education between urban and rural hospitals is wide, as rural hospitals often lack the application of the clinical ladder. This study investigates the effectiveness of using the clinical ladder in a rural Japanese community hospital using the clinical ladder scale and interviews. Through its application, we found that both novice nurses and nursing educators came to recognize the effectiveness and importance of the ladder. However, unfamiliarity with assessments, working conditions, and Japanese culture inhibited the smooth application of the ladder. For the effective application of the clinical ladder, continual training on assessments and the ladder’s effectiveness in clinical situations, along with consideration of educational background, should be enhanced through the monitoring of the clinical ladder.

## 1. Introduction

The clinical ladder is an educational system that indicates the developmental stages of clinical nurses and promotes their growth through individual autonomous behavior and organizational support [1]. The ladder was devised in the 1970s to assess nursing ability in the United States. In Japan, a nursing university hospital first developed it in 1983. It is widely recognized as a system for evaluating individual clinical nurses’ abilities in the hospital nursing setting [2,3]. The clinical ladder evaluates multiple stages of nursing competencies and shows the nurse’s expected skills at each stage as well as the nurse’s ability according to the achievement level [4,5]. By utilizing the clinical ladder, nurses can aim for self-improvement while checking their ability stage. Additionally, it has also been used as a useful tool for human resource development [6].

Japan faces an unprecedented declining birthrate, aging population, and dying society as it moves toward 2025. With the advent of this super-aging society, there is a need for improvement in the quality of nursing for older patients with multiple biopsychosocial problems [7]. Specifically, there is a need to build a nursing care provision system that supports older people to improve their quality of life [8]. As patients age, the variety of pathological conditions due to the coexistence of multiple diseases increases, and the scope of a nurse’s skills needs to expand [9]. The essential nursing practice ability that supports the lives and medical care of various aging patients needs to be strengthened. At the same time, the gap in nursing education between urban and rural hospitals is wide, as rural hospitals often lack the application of the clinical ladder.

In 2016, the Japanese Nursing Association (JNA) established the JNA Ladder. The purpose of the Ladder is to develop and support the educational systems for nursing practice abilities that are common to all nurses, secure and guarantee appropriate assessments of nurses’ skills and development, and advance safe nursing care in patients [10].

The JNA Ladder consists of practical skills that are core nursing skills and five levels of proficiency. The Ladder presents definitions, goals, and actions for each level. In practical application, the concrete content of each action goal has to be defined at each medical institution to fit the clinical environment and care needs. As a first step to broaden rural education, in a previous study, the authors created the “Unnan City Hospital Nurse’s Clinical Ladder” (UNNAN ladder) for use in community hospitals based on the JNA ladder [11]. Although the study ensured the reliability and validity of the UNNAN ladder, there was no correlation between years of experience and age in nursing practice ability, which suggested that independent self-improvement may not lead to an improvement in the nursing practice ability. In addition, in the research on the community hospital’s nurses’ physical skills assessment, clinical experience alone did not appear to improve their knowledge and skills [12].

Because community hospital nurses need to provide thorough nursing for medical treatment after discharge, and for health promotion, disease prevention, and the comprehensive care of older patients [13,14], systematic educational support, like clinical ladders, is needed to improve their practice abilities. However, the initial implementations of clinical ladders in medical institutions have been shown to create not only benefits, but various challenges as well [15]. Yet, the effects and challenges of the implementation of clinical ladders in community hospitals have not been clarified in Japan. Japanese nursing education, which has to heavily deal with an aging society, should have applicability in other international contexts facing potential aging. Thus, the purpose of our study is to clarify the educational effects as well as educators’ and learners’ perceptions of the process through the implementation of the UNNAN ladder in a community hospital.

To this end, we chose a mixed study method that includes both quantitative and qualitative analyses. For the quantitative segment, we performed a descriptive statistical analysis on the items in the UNNAN ladder from the self-assessments that were provided by the nurse learners and those provided by the educators, using 2018 data. We also assessed the reliability of the ladder. For the qualitative segment, we analyzed reflections and reviews on the ladder through responses from the learners and semi-structured interviews with the educators using thematic analysis.

## 2. Materials and Methods

### 2.1. The Setting: Unnan City Hospital

Unnan is a remote rural city in Japan. In March 2020, the total population was 37,637 (18,145 males and 19,492 females), with 39% of it over 65 years. The city has 16 clinics, 12 homecare stations, three visiting nurse stations, and one public hospital [16]. When the study was conducted in 2018, Unnan City Hospital had 281 care beds, 27 physicians, 197 nurses, seven pharmacists, 15 clinical technicians, 37 therapists, four nutritionists, and 34 clerks [12].

The Nursing Department of the Unnan City Hospital has been using the JNA ladder as an index along with the Delphi method (consensus formation method), stipulating specific action content (EPA: Entrustable Professional Activities) for each level. EPA is a core unit of professional practice that can be fully entrusted to a trainee that the trainee has demonstrated the necessary competency to execute the activity unsupervised [17]. The UNNAN ladder, which was created in 2017, had its validity and reliability confirmed at that time [11]. The operation of the postgraduate education system for nursing staff started using the UNNAN ladder in April 2018.

Nurses and midwives are the participants in the UNNAN ladder education program at Unnan City Hospital. The assessment period is from April of one year to March of the following year. The learners (nurses or midwives) carry out systematic education and nursing practice to acquire the practical skills that are necessary for level certification and to complete the training. Practical skills are shown in the assessment table on the UNNAN ladder. The clinical ladder certification committee consists of the nursing department director, the deputy directors, and the educators presenting the necessary training. The certification criteria are that learners’ and educators’ assessments use the UNNAN ladder assessment table and complete the required training criteria.

Two months and nine months, respectively, after starting the program, learners and educators review the program and their learning and educational progress. The educators provide feedback on the learners’ nursing practice and abilities. In the review, the learner’s progress is assessed in terms of goal achievement, and the next goals are set according to the learner’s achievement status. An assessment meeting for level certification is held after January. The assessment meeting participants are learners, educators, and two other evaluators in the same wards. The assessment meeting confirms whether the learner has met the certification criteria and it provides practical feedback. If the learner has not met the certification requirements, then the individual collaborates with educators to create an action plan for the unachieved skills. The assessment committee confirms whether the certification criteria are met and reports this to the Clinical Ladder Certification Committee. Learners who have met the accreditation criteria are level accredited by the Clinical Ladder Accreditation Committee. Level-certified learners continue to study at the next level in the next year, and non-certified learners continue to study at the same level in the next year.

### 2.2. Participants

The ladder has five certification levels, and this study is focused on Level II, novice to independent professional. The research participants comprised 15 learners on the UNNAN ladder Level II in April 2018. The educators were four ward chiefs, and the evaluators were 32 peer nursing staff members and the educators of the nurses’ managers.

### 2.3. Measurements

#### Self-Assessment and Others’ Assessments Using the UNNAN Ladder

The study period was from June 2018 to February 2019. From December 2018 to January 2019, self-assessments and others’ assessments were performed using the UNNAN ladder. From January to February 2019, the assessments were made on a four-point scale from “cannot” to “sufficiently possible” for 66 items on the ladder as self-assessments by learners and assessments by others. Regarding the others’ assessments, each learner was assessed by three evaluators. The results were collected after the assessment meeting. The assessment collection rate was 100% for the 15 participants.

### 2.4. Qualitative and Quantitative Approach

#### 2.4.1. The Review Sheets and Semi-Structured Interviews

Before the meeting reviews between learners and their educators after nine months, the learners were asked to report in advance of these meetings, “what I could do”, “what I could not do”, “improvement points”, and “next steps” on the reference sheets. In the meeting review, the learners and educators held a dialogue while looking at the sheet to clarify future learning content. The educators described the content of the dialogue in the review by adding it to the reference sheet. After the review was completed, the reference sheets were collected, the descriptions were transcribed into an Excel sheet, and coding was performed by the research team. After one-year of the ladder implementation, semi-structured interviews were conducted with the four educators who became evaluators. The interviews were about 40 min. each. The interview content was recorded and transcribed verbatim.

#### 2.4.2. Analyses

The demographic data and scores from the UNNAN ladder were described descriptively. Cronbach’s α coefficient was calculated for each item of self-assessment and others’ assessments, and the inter- and intra-rater reliability were evaluated. A thematic analysis was used to analyze qualitative data to clarify the process of the application of the UNNAN ladder [18]. Researchers carefully read the contents of the reference sheets and semi-structured interviews. One researcher initially coded the content and developed codebooks based on repeated readings using process and concept coding. Another researcher also coded the content and discussed the coding process and codebooks with the first researcher for refinement. During the second coding, the team inducted, merged, deleted, and/or refined concepts and themes by repeatedly referring to the research materials and initial coding. This involved continual discussions until a mutual agreement was reached and no new codes or concepts appeared, thus indicating saturation. These results were also provided to all of the participants, who then provided feedback that was included during a final revision of the themes and concepts. This continued until no new themes emerged, again indicating saturation. Finally, the overarching themes were discussed by the team, and consensus was achieved.

### 2.5. Ethical Considerations

We used items that maintained participants’ anonymity in this research so as to not identify individuals based on their responses. Additionally, we provided information to each participant and deemed the ladder questionnaire’s response as the consent to participate. The obtained data were carefully guarded to avoid the leakage of personal information. This work was only undertaken by the first (SM) and second author (RO). SM was specialized in nursing and medical education and led this study. RO was specialized in medicine and medical education and collaborated with SM in this study. The Unnan City Hospital research ethics review committee approved this research (Approval code 20170019).

## 3. Results

### 3.1. Demographics

As stated, the study included 15 Level II learners. Initially, 17 nurses were targeted, but two had retired by the survey time. Of the 15, six had six years of experience, one had four years of experience, five had three years, and three had two years. After the study period, we interviewed four ward educators, who were deputy head nurses and had become evaluators.

### 3.2. Self-Assessments and Others’ Assessments Results

Table 1 presents the assessment results. The results were collected from the assessment meetings, and the check sheet collection rate was 100%. The average value, standard deviation, minimum value, and maximum value were calculated for each item on the self-assessment and others’ assessment results. Of the 66 items, 24 items on the self-assessments and 10 items on the others’ assessments had an average value of less than three. All 10 items that were less than three on others’ assessments were also less than three on the self-assessments. Cronbach’s α coefficient was calculated for each item of others’ and self-assessments. Cronbach’s α coefficient was 0.8 or higher for all items (see Table 2).

The UNNAN ladder certification criteria were that all assessments by oneself and others should be “possible” and “sufficient”, and that the required training presented should have been taken. Of the participants, four of the nurses with six years of clinical experience met the certification criteria according to the others’ assessments. However, only one of those nurses stated that the certification criteria had been met in the self-assessment. For the nurses with only two years of clinical experience, all three did not meet the certification criteria according to the others’ assessment, while two indicated that they had met the certification criteria according to their self-assessments. Regarding participation in the training, only two nurses with six years of clinical experience met the certification criteria (Table 3).

### 3.3. Perceptions on the Use of the Clinical Ladder

Based on the grounded theory approach, three themes and eight concepts appeared in the comments from learners and educators. The three themes consisted of: (1) understanding the education, (2) limitations of abilities, and (3) drivers for improvement. In applying the ladder, the educators were anxious regarding the insufficient understanding of the ladder and the learners’ abilities to adjust their learning styles effectively. Furthermore, their lack of confidence in assessing learners and adjusting the working conditions for learning impinged on the implementation of the ladder in clinical situations. Ultimately, by implementing the ladder, the educators and learners became used to applying the learning processes in usual clinical situations by realizing the practical application of the educational content to clinical settings and by accustoming to being conscious of the ladder.

#### 3.3.1. Understanding the Education

##### Inadequate Understanding of the Clinical Ladder

At the start of the clinical ladder implementation, educators and learners realized the need for a thorough explanation of its application. Initially, a study session was held for the staff. However, the participants still felt that they did not have a sufficient image of the implementation and they did not fully understand the operational method. At first, educators could not provide adequate learning assistance because they felt that they did not fully understand how to use and apply the clinical ladder. For example, educators stated the following.

*“I didn’t understand it the first time, so I think I wasn’t working enough and talking to them (learners).”*

(Educator A)

*“Learning was delayed, and I was in the last rush.”*

(Educator C)

One learner stated the following.

*“I couldn’t make a plan because I didn’t understand the system.”*

(Learner F)

Because the learners had insufficient understanding and experience with the clinical ladder, initially, they could not systematically study its content while learning it. Thus, educators and learners were both confused by the new system when the ladder was introduced.

##### Varieties of Learners and Situations

The educators found it difficult because the learners did not have independent learning attitudes. They also found it challenging to teach according to the natures and attitudes of different learners. They were confused by trying to balance learner diversity and advancing learners on the clinical ladder. The learners also had difficulty adjusting to the learning environment in the clinical situations that they were placed in. For example, some educators pointed out the following.

*“Everyone is quiet. I don’t have much reaction, so how should I get involved. Sometimes I’m just talking.”*

(Educator D)

*“There was no response when I proposed a day that was convenient for attending the workshop by comparing it with the roster.”*

(Educator A)

*“We were all positive and honest people, so I asked what this meant and what should I do to do this?”*

(Educator C)

One learner made the following comment.

*“I wasn’t able to make any progress on my learning because I was put off.”*

(Learner C)

Educators also stated the following.

*“On the other hand, I also realized that the learners were growing and that considering the learners’ diversity could lead to improved learning.”*

(Educator B)

*“When I evaluated it, the second and third years were different, so I thought that everyone was growing up.”*

(Educator A)

One learner stated the following.

*“I think I can participate in the in-hospital training and use it in clinical practice little by little.”*

(Learner K)

In the process of supporting learners, educators deepened their understanding of learners and saw that there were new factors to be considered. Although they faced difficulties from inappropriate attitudes and negativity, they also saw positive attitudes and personal growth. The educators confirmed that the learners gained experience and improved their ability to practice nursing through the process.

##### Opaque Learning Process

The educators found it difficult to provide concrete support for the learning environment while respecting learners. Although the learners’ independence was important, the educators felt the importance of their role as coordinators, such as the need for creating plans and providing early support. For example, educators made the following comments:

*“When I called out, I was often told that ‘I didn’t do it’, so I didn’t say anything.”*

(Educator D)

*“I couldn’t confirm each learner’s process on the way. I couldn’t balance how much to call and whether to leave it to the person’s pace and intention.”*

(Educator C)

Some of the comments from learners’ were as follows.

*“I should have been careful about the training day and made hopes myself.”*

(Learner E)

*“It would have been nice to have time to work on it, such as deciding when to watch the video training.”*

(Learner A)

The educators felt that their efforts for planned learning and their planning were insufficient due to a lack of learning experience using the ladder. However, the educator’s role was to offer specific advice and assistance to learners in daily nursing care, so that learners could develop an attitude of learning for themselves and consider what they should do to improve. For example, educators stated the following.

*“Since the parts that are made and the parts that cannot be made become clear, I can say that we should focus on the parts that are not made. I can teach them what to look for in informed consent.”*

(Educator B)

*“It’s natural for me to be able to do this in the third year, but I can’t do it, etc. So I think I have to experience it, and I sometimes let learners experience it.”*

(Educator D)

The educators wanted an intervention that respected the learner’s independence. Although they felt the need to support the learning environment, they were confused because they could not intervene appropriately. The educators were looking back and trying to come up with concrete ways to act in order to provide better support.

#### 3.3.2. Limitation of Abilities

##### Reliable Assessment of Others

The educators felt that it was vital for them to understand the learners when making assessments. They emphasized that they could directly confirm the learners’ practical abilities, even in a busy daily work situation. However, they had minimal experience in the evaluator position, so they worried about their assessments. For example, they stated the following.

*“I have a reflection that I haven’t seen the person. I didn’t know how to evaluate it.”*

(Educator D)

*“To be honest, I didn’t focus on the people who were the target of the ladder every day, so I didn’t know what the learners could and couldn’t do.”*

(Educator A)

Additionally, the educators worried about whether they were able to evaluate correctly. For example, they made the following comments.

*“Isn’t the assessment standard just my subjectivity? I didn’t see it properly, so whether the assessment was appropriate or not.”*

(Educator B)

*“I felt that if I just looked at the learners from my perspective at the individual level, the demands would be different.”*

(Educator D)

As part of the ladder’s introduction, for educators, there were no opportunities for acquiring the knowledge and skills to evaluate learners. Although assessments by objective management were conducted yearly, no special training was given regarding how to conduct the assessment. The awareness that they had to evaluate direct practical ability reasonably led to anxiety about the assessment.

##### Difficulties from Overwork

Assessment meetings were sometimes held outside working hours. This meant that educators struggled to coordinate with each other’s schedules and hold assessment meetings during their busy work weeks. They also found it challenging to complete their work during working hours to facilitate the ladder. Further, they were worried about their management and what to do if the number of learners increased in the future. For example, the following comments were made.

*“I took it home and evaluated it.”*

(Educator C)

*“It’s sporadic that there are three evaluators at the assessment meeting, so I made time at midnight or something like that.”*

(Educator A)

*“To hold an assessment meeting, it was difficult to create a roster with an awareness of working together.”*

(Educator D)

*“I thought it was impossible during work time. I think it should be during work time, but I can’t do it.”*

(Educator B)

*“This time, there were only two learners, but if the number of learners increases in the future, I think it will not be possible.”*

(Educator A)

One learner stated the following.

*“As for my learning, I was so busy with work that I had to put it off and didn’t make any progress.”*

(Learner C)

Learners also reflected on the difficulty of making time and advancing their learning during their busy daily work. Moreover, the learner’s assessment meeting was an addition to the educators’ workload; they found it difficult to secure time for this new work in their daily work schedule. Although they tried to solve time difficulties by making adjustments when creating the roster, it was challenging to finish within business hours.

#### 3.3.3. Drivers for Improvement

##### Fruitful Review

Before starting the ladder system, educators already had perceptions that were based on the existing personnel assessment system within the community hospital. In that process, they were supposed to confirm a learner’s individual annual goals and advise on achievements in the term-end reviews. This time, with the operation of the clinical ladder, they realized that the reviews were more fulfilling because the purpose of the assessment and its contents were clearer. For example, the educators made the following points.

*“The reviews were straightforward, and the content was deepened. It became an index for the reviews.”*

(Educator A)

*“In the reviews, the three of us were able to tell them that we think this is good enough.”*

(Educator B)

Among the learners’ the following comments were made.

*“I was able to create more opportunities to think about my learning than ever before.”*

(Learner I)

*“I have more opportunities to communicate with my seniors in the ward, and my motivation for learning has increased.”*

(Learner D)

The educators could advise, approve, and accept learners by conducting reviews with a clear purpose, as stipulated in the clinical ladder. The clarification of the criteria for reviews based on the ladder led to increased motivation for their assessments.

##### Contribution to Clinical Situations through Learning

The educators recognized that there was a specific effect from attending training, which was a requirement for level certification. They were particularly aware of the impact of facilitating learning by establishing particular criteria. However, they felt that various training styles were dependent on the learner. The appropriate assessment method and the effects of learning to be completed in the field remained unclear. For example, the educators made statements, as follows.

*“The people here were earnest about the training and were able to understand it all.”*

(Educator B)

*“There was also an item that learners would not have learned without the training quota.”*

(Educator A)

*“Learning is done properly with tests, but sometimes it’s hard to see if they are making use of it.”*

(Educator C)

*“Since the training was carried out in a hurry in the latter half of the curriculum, I don’t know if the learners were learning.”*

(Educator D)

However, the educators continued to recognize the improvement in the learner’s education from the clinical ladder and, in particular, the expected future growth among the young learners with less than six years of experience. Furthermore, for the educators, the results of individual learning and the synergistic effect of improving the connection between learners and their attitude toward learning reflected an improvement in the learning motivation and quality of care of the organization. For example, educators made the following remarks.

*“Young people will grow steadily, so it is important to adjust learning opportunities in the future.”*

(Educator A)

*“I hope that we can make horizontal connections by advancing this education system. The feeling of learning together is wonderful.”*

(Educator B)

*“It would be great if I could make use of it in my work as I proceeded with learning. I think that a tendency is gradually emerging. I hope that once I learn it, I can feel that I will use it more thoroughly, and I can contribute to the organization’s growth.”*

(Educator D)

The learners were motivated by the need to clarify their own goals and adapt their learning content to the actual field situation. For example, they made the following comments.

*“It’s interesting to be able to put into practice what I’ve learned. I want to make more effort so that I can put what I’ve learned into practice.”*

(Learner J)

*“I want to clarify what kind of nurse I want to be, and I think that will lead to patient care.”*

(Learner M)

##### Creating an Educational Culture

The use of the UNNAN ladder as a new system was the first experience for the entire hospital, and many were confused due to their unfamiliarity with it. However, the educators were motivated to facilitate and habituate future assessment methods, citing that the tree education system had not been incorporated into their own training. For example, the educators stated the following.

*“I haven’t been able to incorporate the curriculum for myself, so I’ve been looking at the flow chart many times. I have to understand more. It’s important to feel that education is the norm.”*

(Educator D)

*“I didn’t know the flow of the curriculum, so I was confused about what to do. It’s not good. I want to proceed smoothly in the future.”*

(Educator A)

The educators realized that the curriculum had just begun, and that they were unfamiliar with the ladder’s use, which took some time to confirm. Further, this meant that their actions regarding the ladder were delayed and did not go smoothly. However, the following point was made regarding the improvement.

*“Last year was late, but this year I got a self-assessment early, and I learned about individual characteristics and goals early enough, which proceeded smoothly.”*

(Educator C)

The reflections among the learners’ included the following.

*“I would like to self-check my level of understanding while checking what I have learned and what I have not learned. It would be great if I could make a difference as a matter of course.”*

(Learner K)

*“I would like to make work adjustments so that I can participate in in-hospital training on a habitual basis, and I hope there is a system that can return the content to the hospital.”*

(Learner B)

As stated, the educators were initially confused by what was unfamiliar to them. Still, after a year, they were conscious of a new motivation and making a habit of taking advantage of last year’s experience. Additionally, the learners became conscious of controlling the content of their learning, devising new ways of learning, and adjusting the content for the future after one year of experience with the ladder.

## 4. Discussion

This study clarified the improvement of learning through the application of a clinical ladder along with the difficulties that are felt by learners and educators during the implementation process in the context of Japan. The educators were able to deepen their understanding of learners by consciously being involved in their learning support. Moreover, the process was effective in enhancing the reviews and reflections based on the clinical ladder. The educators were worried about evaluating others, but there was no significant difference between the learners’ self-assessments and the others’ assessments, which implied that these were appropriate. However, educators and learners had difficulty due to a lack of understanding of the system, a lack of experience among the educators, and time difficulties in addressing the clinical ladder system as a new task. Notably, it became clear that educators recognized the value of the clinical ladder, saw it as having a learning effect that could be returned to the organization, and expected it to continue becoming part of the hospital culture.

On the others’ assessments, nurses with six years of clinical experience met the certification criteria more often than nurses with two years of clinical experience at the end of the study period. In the process of consciously providing learning support, educators realized that practical abilities improved with the number of years of experience. Regarding self-assessments, two of the three second-year nurses felt that they met the certification criteria, positively evaluating their nursing practice abilities. In Japanese culture, “modesty” is generally an ingrained aspect of learning style [19]; thus, nursing professionals may have low self-efficacy in rural hospitals [20]. Moreover, many learners may not be accustomed to evaluating themselves; therefore, the quality of the self-assessment may improve as the learning experience in the field increases.

In this study, we found that learners who may not have had self-assessment abilities appeared to overestimate their own abilities in some instances [21]. However, of the 66 UNNAN ladder items, 24 items in the self-assessments had an average of less than three, while only 10 items in others’ assessments had that average, which meant that, in such cases, learners tended to underestimate themselves. Certification criteria were established for each item on the “others’ assessments”, “self-assessments”, and “attendance of training.” In particular, nurses with six years of clinical experience reflected low self-assessments, even if they were certified by the others’ assessments. As a characteristic of Japanese culture, there is a tendency to provide a negative or low evaluation of oneself [22].

Notably, our research found that, from a Japanese learning culture perspective, looking at a future assessment system for the ladder, the comprehensive self-assessments reflected that those with high abilities under-evaluated themselves, while those who were inexperienced over-evaluated themselves [1,2]. The implication is that, going forward, learners should gain experience and familiarity with self-assessment.

We found that the learners were able to apply their learning through the clinical ladder to clinical practice. In addition to confirming the effects of learning, the educator’s role was found to be significant in terms of staff management [23,24]. The educators could effectively enhance the reviews based on the personnel assessment system [25]. Before starting the ladder system, reviews were conducted based on the personnel assessment system [26]. In the review, the individual’s annual goals were confirmed and, in the follow-up review, advice was given regarding how the results and achievements were to be accomplished [26]. The educators could probably provide specific guidance and assistance by conducting the review using the ladder items as an index and, thereby, achieve a more effective review. For learners, the problems and solutions in nursing practice could be addressed and confirmed together with their manager, and the review could, therefore, be more meaningful.

The educators indicated that they found it challenging to evaluate others. They were anxious about their assessments, but there was no significant difference between the learners’ self-assessments and others’ assessments. One reason why the educators felt anxious about assessing learners was that they were unfamiliar with an assessment system, like the clinical ladder. There were few opportunities to assess others where systematic assessment was done. Although the educators expressed a lack of experience in evaluation, Cronbach’s α coefficient was 0.8 or higher for each item on the self-assessments and the others’ assessments, with the reliability confirmed. The 10 items from the others’ assessments with an average assessment value of less than three were included in the 24 items with an average self-assessment value of less than three in the self-assessments.

The educators tended to emphasize accuracy by directly assessing the learner’s progress, grasping the degree of learning practice, and the multifaceted learner’s understanding. In this study, all of the assessors were educators, deputy educators, and department managers. Nursing managers placed importance on building relationships of trust with others and on collaborating [25,26]; thus, the expectation was that they had a strong desire for a more accurate assessment.

For the evaluators to make an appropriate assessment with confidence, a common understanding of the assessment process needs to be established within the organization by providing training on both the educational and assessment methods [3,5]. In Japan, as the cultural background is one where humility is a virtue [19], this needs to be considered when the mechanism is created. A future task is to ensure that educators are able to evaluate with confidence. Therefore, there is a need for greater education on the assessments of learners to improve accuracy and reduce the educators’ burden.

The educators also struggled with time management in applying the clinical ladder, and in securing the time for added tasks, such as offering support and holding assessment meetings within working hours. Nurses’ working hours remain a problem, and it is necessary to reduce this burden to avoid injuries from overwork and ensure medical safety [27].

Additionally, the educators were confused, as they felt that they were giving ineffective support. Some possible reasons for this were a lack of experience as coordinators of learning support and the emphasis on support that respected independence [28]. In addition, educators pointing to the difficulty of offering support based on an insufficient understanding of the system. Although the educators recognized that the UNNAN ladder could become a cultural fixture for the organization, contributing to effective nursing education, they remained confused about the unfamiliar educational methods and struggled with the business management that is required in the academic environment [29]. When the clinical ladder was initially introduced, both the educators and the learners may have delayed trying to use the system due to an insufficient understanding of it.

It is critical to recognize the need for the clinical ladder and understand it as a system of learning to ensure its continuity and effectiveness [2,3,6]. The educators expected to see significant learning effects from the ladder that could be returned to the organization as well as individual learning, such as synergistic effects through multiple learners, which could provide mutual benefits among nurses.

Clinical ladders can improve the quality of nursing throughout the workplace by supporting the learning of individual nurses and creating an environment where they can learn from each other [30]. In the future, community hospitals need to provide educators and learners with continuous information on educational methods in order to introduce clinical ladders smoothly. To that end, these institutions need to position nursing education as one of the significant pillars of organizational management with the organization working together on the mechanism [31].

This study has the following limitations. The first one is the study’s setting. This study was performed in a single rural community hospital, which reduces its external validity and transferability. However, as medical conditions in Japan are similar to those faced in other Asian countries because of aging, our result can be used as a reference for nursing educational systems in other countries. This study’s duration and sample size are the second. This study followed up with the participants for about one year, which might not be sufficient to observe the changes in the participants and the effective application of the educational system and through four educators’ interviews. Future studies could adopt a longitudinal design to confirm the effectiveness of the clinical ladder with variable educators. Third, the study used a mixed method for assessing the participants’ challenges in the educational system in order to identify the areas for improvement of the application of the clinical ladder. Future studies could also look at larger sample sizes to apply other methodological analyses.

## 5. Conclusions

This study clarifies the practical application of the clinical ladder in a community hospital in Japan. The application of the clinical ladder has the possibility to effectively improve nursing skills through collaboration among educators and trainees. In the process of applying the ladder, the educators and learners struggled with changes in their educational situations and perceptions that are affected by Japanese culture. To mitigate these challenges, continual training on educational assessments in clinical situations should be enhanced by monitoring the effectiveness of the clinical ladder.

## Figures and Tables

**Table 1:** The results of self- and others’ assessments on the “Unnan City Hospital Nurse’s Clinical Ladder” (UNNAN ladder).

					Self-Assessment *n* = 15				Others’ Assessment *n* = 32			
Competency	Explanation	Subcategory	No.	EPA	Average	SD	Min	Max	Average	SD	Min	Max
The ability to capture needs (A)	Get advice and capture the needs of recipients and situations (places) of care	A1 Gather information independently from the physical, mental, social and spiritual aspects necessary for the recipient of care	1	Build relationships of trust with patients	3.40	0.51	3	4	3.28	0.51	2	4
			2	Perform physical examination independently	3.13	0.64	2	4	3.19	0.71	2	4
			3	Record the contents of the physical assessment	3.13	0.64	2	4	3.22	0.68	2	4
			4	Point out abnormalities in physical assessment	3.07	0.59	2	4	3.14	0.68	2	4
			5	Gather information about the mental aspects of patients	2.87	0.52	2	4	3.06	0.53	2	4
			6	Gather information from patients’ family	2.93	0.70	2	4	3.11	0.57	2	4
			7	Gather information on patient’s illness	3.13	0.64	2	4	3.22	0.68	2	4
			8	Understand how patients think about their diseases	2.93	0.46	2	4	3.03	0.61	2	4
			9	Recollect information in requiring additional information	3.13	0.52	2	4	3.17	0.61	2	4
			10	Considering logically when gathering information	3.27	0.59	2	4	3.19	0.40	3	4
		A2 Grasp the problem as the whole image of the recipient of care based on the obtained information	1	Extract physical problems	3.07	0.59	2	4	3.25	0.69	2	4
			2	Extract psychological problems	3.00	0.53	2	4	3.06	0.58	2	4
			3	Extract social problems	2.93	0.59	2	4	**2.92**	0.55	1	4
			4	Understand and assess patient’s symptoms	3.07	0.59	2	4	3.14	0.68	2	4
			5	understand the treatment policy of physicians	2.87	0.52	2	4	3.25	0.65	2	4
			6	Consider complaints and observations of patients and families when planning a nursing care plan	2.80	0.68	2	4	2.97	0.51	2	4
			7	Understand individuality and can plan nursing care	2.67	0.62	2	4	2.97	0.38	2	4
			8	describe the necessary procedures for nursing in nursing information	3.13	0.64	2	4	3.36	0.59	2	4
the ability to care (B)	Practice nursing according to care recipients and situation (place)	B1 Practice nursing based on standard nursing care plan with consideration of the individuality of recipients of care	1	Express a calm look for easy speaking	3.40	0.51	3	4	3.64	0.49	3	4
			2	Practice care with the respect for individuality of patients	3.07	0.59	2	4	3.19	0.40	3	4
			3	Practice standard care and techniques	3.13	0.64	2	4	3.22	0.64	2	4
			4	Perform standard procedures, treatments, and countermeasures according to the manual	3.00	0.53	2	4	3.28	0.45	3	4
			5	Understand handling of infectious diseases and respond	3.00	0.65	2	4	3.17	0.45	2	4
			6	Follow the treatment policy and do the correct medical treatment	3.07	0.46	2	4	3.31	0.58	2	4
			7	Go actively to the patient’s bedside	3.40	0.63	2	4	3.33	0.53	2	4
			8	understand the complications associated with diseases	2.80	0.56	2	4	2.94	0.53	2	4
			9	Use properly instruction for abnormal situations of patients	3.13	0.64	2	4	3.19	0.62	2	4
			10	Do nursing such as clean care etc. which is missing considering patients’ ADL	3.13	0.52	2	4	3.19	0.62	2	4
			11	Prioritize care	3.20	0.56	2	4	3.03	0.77	1	4
			12	Record nursing practice accurately	3.20	0.68	2	4	3.31	0.62	2	4
			13	Practice care of acute patients	2.80	0.77	1	4	2.92	0.73	1	4
			14	Practice care of seriously ill patients	2.73	0.70	1	4	2.89	0.71	1	4
		B2 Get the information you needed for practicing care for recipients	1	Gather the necessary information from the medical record to know the progress	3.13	0.64	2	4	3.31	0.62	2	4
			2	Check patient information necessary for care practice	3.07	0.59	2	4	3.17	0.61	2	4
			3	Organize information to evaluate the effect of care	2.93	0.46	2	4	3.00	0.68	1	4
			4	Collect information and instruments necessary for practicing nursing care planning	2.93	0.46	2	4	3.11	0.57	2	4
			5	Ask actively for guidance on inexperienced nursing skills	3.13	0.52	2	4	3.33	0.53	2	4
			6	Check the unclear part of the doctor’s instructions	2.93	0.46	2	4	3.33	0.59	2	4
			7	Report the results of physical assessment	3.07	0.59	2	4	3.22	0.59	2	4
			8	report reactions accurately by nursing care	2.93	0.46	2	4	3.19	0.58	2	4
			9	Evaluate practiced care based on nursing care plan	2.87	0.52	2	4	3.11	0.57	2	4
		B3 Provide assistance according to the circumstances of care recipients	1	Understand changes in patient needs	2.93	0.46	2	4	2.97	0.45	2	4
			2	Advise patients through nursing care	2.80	0.56	2	4	3.08	0.50	2	4
			3	Provide necessary information according to inspection purpose	3.00	0.53	2	4	3.14	0.59	2	4
			4	Provide nursing technical assistance fitting patients based on treatment plans	2.93	0.46	2	4	3.17	0.56	2	4
			5	Evaluate pressure ulcer properly	2.87	0.52	2	4	3.14	0.72	1	4
			6	Provide an appropriate therapeutic environment for pressure ulcer patients	2.93	0.46	2	4	3.03	0.65	1	4
			7	Adjust environment according to patients	3.07	0.46	2	4	3.03	0.51	2	4
			8	Help with physical pains	3.00	0.53	2	4	3.17	0.56	2	4
			9	Act with responsibility according to instructions in emergency situations	2.80	0.77	1	4	2.97	0.84	1	4
			10	Demand appropriate support in emergency situations	2.80	0.68	1	4	3.06	0.79	1	4
the ability to collaborate (C)	Identify stakeholders necessary for nursing development and exchange information	C1 Understand the differences in positions and roles of stakeholders who surround the care recipients and can actively exchange information with them	1	Share information with the nursing team	3.40	0.63	2	4	3.22	0.54	2	4
			2	Report the information I got to the team leader	3.47	0.52	3	4	3.33	0.59	2	4
			3	Report the information I got to the ward leader	3.40	0.63	2	4	3.22	0.59	2	4
			4	Contact the nursing team to consult the necessary information	3.40	0.63	2	4	3.25	0.55	2	4
		C2 Communicate closely with stakeholders	1	Be conscious of team play	3.33	0.62	2	4	3.06	0.58	1	4
			2	Summarize the necessary information and transmit it	3.07	0.80	1	4	3.11	0.67	1	4
			3	Consult with stakeholders in getting stuck	3.40	0.63	2	4	3.08	0.65	1	4
		C4 Grasp the direction of nursing and the situation of stakeholders and exchange information	1	Provide the obtained information to the chief and the counselor	3.27	0.59	2	4	3.19	0.67	1	4
			2	Provide information to patients, doctors, and nursing teams	3.13	0.64	2	4	3.17	0.61	2	4
			3	acknowledge the direction of the patient	3.07	0.88	1	4	3.08	0.73	1	4
			4	Write a nursing summary	3.27	0.59	2	4	3.28	0.51	2	4
the ability to support decision-making (D)	Make use of the attitude of care recipients and people around you for nursing	D1 check the thoughts, thoughts, and hopes of recipients of care and people around them intentionally	1	Attend informed consents and record the degree of understanding and response of patients and their families	2.73	0.96	1	4	2.81	0.82	1	4
		D2 Relate patient’s thoughts, ideas and hope to care	1	Inform the nursing team about the wishes of patients and their families	3.20	0.56	2	4	3.08	0.55	2	4
			2	Provide nursing practices tailored to the needs of patient and their families	3.13	0.64	2	4	3.08	0.55	2	4
			3	Change nursing plan based on the needs of patient and their families	2.87	0.74	2	4	2.94	0.71	1	4

SD: standard deviation, EPA: Entrustable professional activity.

**Table 2:** Cronbach’s α coefficient for each scale of the UNNAN ladder.

	Self-Assessment	Others’ Assessments
A1	0.95	0.92
A2	0.95	0.93
B1	0.95	0.93
B2	0.94	0.94
B3	0.96	0.95
C1	0.99	0.93
C2	0.88	0.83
C4	0.93	0.84
D1	1.00	1.00
D2	0.87	0.85

**Table 3:** The results of the UNNAN ladder certification assessment.

		Others’ Assessments		Self-Assessments		Participation in Seminars	
Clinical Experience	*n*	Certified	%	Certified	%	Certified	%
6 years	6	4	67%	1	17%	2	33%
4 years	1	0	0%	0	0%	1	100%
3 years	5	2	40%	3	60%	3	60%
2 years	3	0	0%	2	67%	0	0%

## Data Availability

All relevant datasets in this study are described in the manuscript.
